# Stand Up for Yourself: Tackling Sedentary Behavior through Exercise and Lifestyle

**DOI:** 10.3390/ijerph20054673

**Published:** 2023-03-06

**Authors:** Rogério César Fermino, Paulo Henrique Guerra

**Affiliations:** 1 Research Group on Environment, Physical Activity, and Health, Postgraduate Program in Physical Education, Federal University of Technology-Paraná, Curitiba 81310-900, Brazil; 2 Postgraduate Program in Physical Education, Federal University of Paraná, Curitiba 81531-980, Brazil; 3 School of Medicine, Federal University of Fronteira Sul, Chapecó 89815-899, Brazil

Because of their direct relations to the process of health and disease throughout life, physical activity and sedentary behavior emerge as priorities in the global public health agenda [1,2]. If there is no reduction in physical inactivity worldwide, around 500 million new non-communicable chronic diseases could occur by 2030, with most of these in low- and middle-income countries [3].

The available evidence supports the understanding that a lack of physical activity and substantial periods of sedentary behavior is associated with an increased risk of cardiovascular disease, cancer, and premature mortality [4,5]. Non-communicable chronic diseases show increasing trends globally, resulting in high morbidity and mortality rates and high costs to health systems [3,6].

The COVID-19 pandemic decreased physical activity and increased sedentary behavior [7]. Most people were confined to their homes for months and exposed to less energy-intensive activities [7]. From a global perspective, the research contributes to the broad understanding of developing interventions that reduce physical inactivity and sedentary behavior [2]. How can we make it easier for people to move around in their leisure time, during commutes, when attending work/school/university, or staying at home?

Physical activity and sedentary behavior are multidetermined and require complex approaches and efforts for confrontation [8,9]. Thus, this *IJERPH* Special Issue, “Stand Up For Yourself! Tackling Sedentary Behavior Through Exercise and Lifestyle Change”, invites researchers worldwide to share evidence and discuss the main determinants and strategies of interventions from an interdisciplinary perspective.
